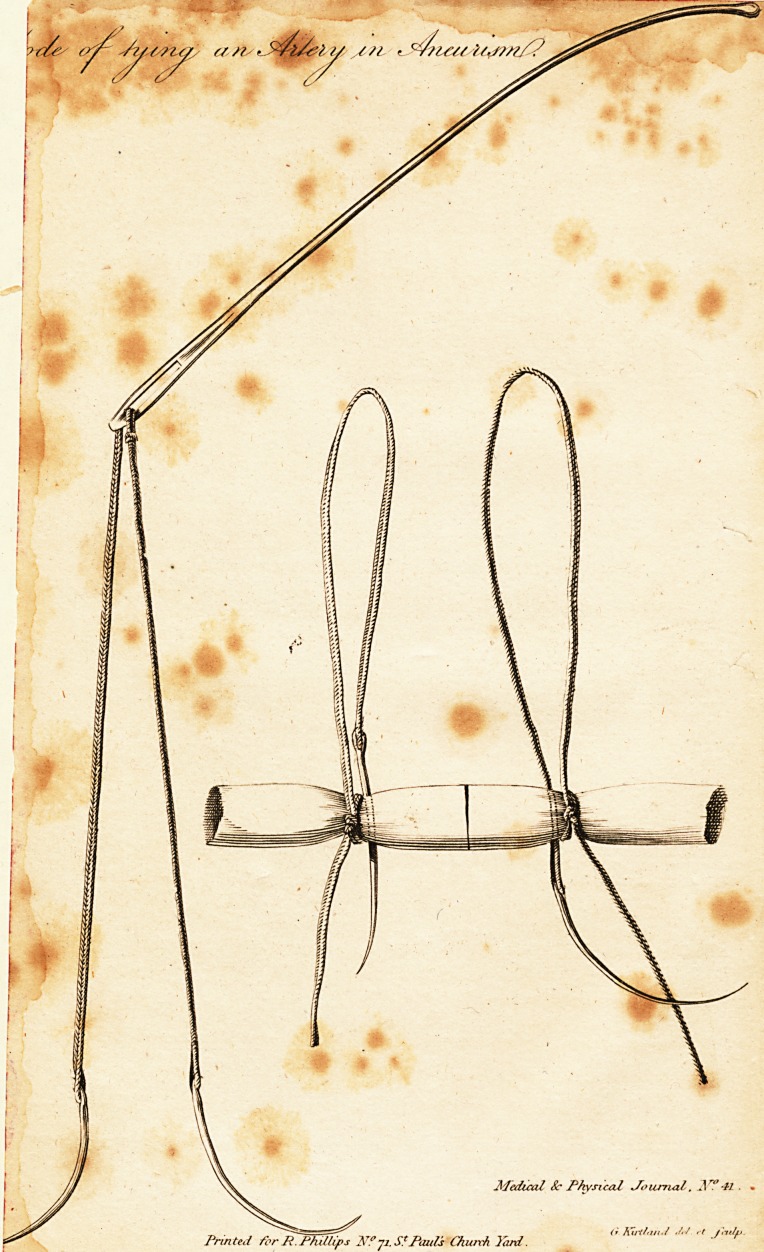# Mr. Cooper, on Aneurism

**Published:** 1802-07

**Authors:** Astley Cooper


Medical & Physical Journal, No. 41.

Printed for R. Phillips No. 71, St. Paul's Church Yard. G. Kirtland del. et sculp.

THE

### Medical and Physical Journal.

VOL. VIII.] JULY, 1802. [NO. XLI.

### To Dr. BRADLEY.

DEAR SIR,

THE following cases of Aneurism contain some facts relative to the operation for that disease, which appear to me to deserve attention; and I shall therefore request you to insert them in the next Number of your Journal, as from the extent of its circulation, they are thus certain of being generally known.

I am, &c.

*June* 24, 1802. ASTLEY COOPER.

---

The alteration which has been made by my friend Mr. Abernethy, in Mr. Hunter's operation for popliteal Aneurism, by tying the artery with two ligatures instead of one, and afterwards dividing the vessel,* has certainly tended to lessen the danger of hæmorrhage, at the time the ligature is occasioning ulceration in the coats of the artery. But there is a danger resulting from this practice, the nature of which the first of the following cases will explain, and the others will shew the mode which has been adopted to prevent such accidents in future.

### CASE I.

Edward Powell, aged 27, by trade a rope-maker, was admitted into Guy's Hospital on the 7th of April for a popliteal Aneurism. It appeared that he had been a healthy, laborious man until November last, when he first felt, whilst at work, a sense of stiffness in his knee, with a considerable degree of pain, and perceived a small swelling in the ham, which, he said, did not then pulsate.

He continued to work, though with difficulty, for three weeks, complaining of great pain in every attempt to straight-

---

* See Abernethy's Essays, Part iii. also a work of Mr. Maunoir, Surgeon at Geneva, and Mr. John Bell's Surgery, Vol. i.

en the leg; and the ſwelling increaſing, and becoming pulſatory, he obtained admiſſion into the London Hoſpital.

Prior to any operation being propoſed, an ingeniouſly conſtructed inſtrument was applied by Mr. Blizard, whoſe patient he was, with a hope of obliterating the femoral artery by compreſſion.

The points of ſupport for this inſtrument were the outer part of the knee and the great trochanter, a piece of ſteel paſſing from the one to the other; and to the middle of this a ſemicircular piece of iron was fixed, which projected over the femoral artery, having a pad at its end moved by a ſcrew, by turning which, the artery was readily compreſſed, and the pulſation in the Aneuriſm ſtopped, without any interruption to the circulation in the ſmaller veſſels.

But although this patient poſſeſſed unuſual fortitude of mind, and indifference to pain, he was incapable of ſupporting the preſſure of the inſtrument longer than nine hours; and when it was looſened, the pulſation in the tumour returned with unabated force.

After a fair trial of this plan, the man quitted the hoſpital, and placed himſelf under the care of Mr. Young, ſurgeon, in Coleman Street, who requeſted me to admit him into Guy's Hoſpital.

At the time of his admiſſion, the tumour in the ham was large, and had a ſtrong pulſation, but the leg was free from ſwelling; and the man's general health appearing to be tolerably good, the operation for Aneuriſm was propoſed to him, and it was performed on the ſixth day from his admiſſion, in the following manner.

An inciſion, three inches in length, being made in the middle of the inner part of the thigh, expoſed the ſartorious muſcle, which being gently raiſed by the finger brought the femoral artery into view. The artery being then ſeparated from the vein and nerve, and detached from the ſurrounding parts, an eyed probe armed with a double ligature was conveyed under it; the probe being cut away left two ligatures beneath the veſſel: the threads were then ſeparated and the one tyed upon the upper, the other upon the lower portion of the artery, with ſuch a degree of force as could be uſed without the riſk of cutting its coats. The veſſel was then divided between the ligatures by means of a probe-pointed biſtoury, and the operation was ſuppoſed to be concluded.

But as I was proceeding to dreſs the wound, I ſaw a ſtream of blood iſſuing from the artery; and when the blood was ſponged away, one of the ligatures was found detached from the veſſel. Soon after, the other alſo was forced off, and thus the divided femoral femoral artery was left without a ligature; and unleſs immediate aſſiſtance had been afforded him, the patient muſt have periſhed under hæmorrhage.

No apprehenſion was excited of the caſe terminating fatally, becauſe I knew that the bleeding was completely in my power, and putting my fingers therefore on the groin, and compreſſing the artery upon the pubis, the divided veſſel ceaſed to bleed.*

I then directed an aſſiſtant to preſerve a ſimilar preſſure upon the groin, whilſt I attempted to ſecure the artery. The divided veſſel being conſiderably retracted under the ſkin, it was with ſome difficulty drawn out by a tenaculam, and ſecured by a needle and ligature.

The delay occaſioned by this circumſtance produced no bad conſequences; the man had but little ſymptomatic fever. On the twenty-fourth day after the operation, the ligatures came away; and on the 20th of May, he was diſcharged the hoſpital, as it was thought that his health was ſuffering from remaining in it; but it was found that the conſtitutional irritation which then appeared, had ariſen from the coagulum in the aneuriſmal ſac having become diſſolved and putreſcent, and it afterwards diſcharged itſelf by producing an abſceſs in his ham. The wound in the thigh was healed on the 25th of May.

It was a very fortunate circumſtance that the ligatures were forced from the veſſel whilſt the man was ſtill in the operating theatre, as, if he had been put to bed, and quitted, he would have fallen a victim to bleeding before any aſſiſtance could have been procured.

Had this accident happened only to myſelf, I ſhould have rather attributed it to ſome fault in my mode of performing the operation, than to any want of ſecurity in the operation itſelf; but when I ſtate that the ſame accident has happened to Mr. Cline, whoſe ſuperior ſkill and caution are univerſally acknowledged, it muſt be confeſſed by every candid mind, that it is one againſt which it is neceſſary to guard, and to which all may be occaſionally liable.

Mr. Cline, in the laſt autumn, performed the operation in the manner I have deſcribed; he uſed extraordinary pains in ſecuring the ligatures, which were placed upon the artery an inch aſunder; the artery was then divided, the wound dreſſed, and the patient put to bed. In three hours a violent hæmorrhage ſucceeded; one of the dreſſers happened fortunately to be

 upon

* It is a circumſtance which, perhaps, may not be generally known, that it is not difficult to ſtop any bleeding in the thigh, or leg, by preſſure upon the femoral artery as it paſſes over the pubis. In an amputation of the thigh, performed by Mr. Lucas, it was neceſſary to remove it ſo near to the groin, that no tourniquet could be applied, and the patient was ſecured from bleeding during the operation, by preſſure upon the artery at the groin.

upon the ſpot, preſſed upon the artery,[a] and ſent for Mr. Cline, who, when he removed the dreſſings, found the upper ligature lying looſe in the wound. He ſecured the artery by means of a needle and ligature, and the man did well.

Theſe accidents naturally gave riſe to reflections upon the means which were to be employed to obviate them; and the firſt which ſuggeſted itſelf was to include a larger portion of artery between the two ligatures: But this plan was given up, when it was recollected that many branches of arteries muſt be divided, and that it was a mode of ſecurity (if it was ſo) which could only apply to particular caſes of Aneuriſm; ſince, in ſome ſituations of that diſeaſe, there is ſcarcely any length of veſſel between the tumour and a principal anaſtomoſing branch of the artery.

A plan of greater ſecurity and more general application conſiſts in conveying the ligatures by means of two blunt needles under the artery, an inch aſunder, and cloſe to the coats of the veſſel, excluding the vein and nerve, but paſſing the threads through the cellular membrane ſurrounding the artery. When theſe are tied, and the artery is divided between them, the ligatures will be prevented from ſlipping from the artery by the cellular membrane through which they are paſſed. In this way I lately performed the operation in the following caſe.

### Case II.

———, aged 19 years, was wounded in the brachial artery in bleeding. The artery, I was informed, was at the time of the operation ſuſpected to have been punctured, from the florid colour of the blood, the force with which it iſſued, and from the pulſatory motion of the ſtream. A very tight bandage was applied, but the lad ſuffered ſo much pain, that it became neceſſary to looſen it in about eight hours; and the bleeding did not recur.

In two months he returned to the place at which the accident happened, with a pulſatory ſwelling at the fore part of the elbow joint, under the ſcar which had been made in bleeding. Nine weeks after the accident, I was requeſted to ſee him.

The ſwelling was then about the ſize of a pigeon's egg, it pulſated ſtrongly; and as the diſeaſe was gradually increaſing, it became neceſſary to perform the operation for Aneuriſm. I made an inciſion upon the brachial artery, three inches above the ſwelling, and paſſed a ligature by means of a blunt needle, cloſe to the artery, excluding the vein and the median nerve, with which this veſſel is accompanied; and having tyed it, another was paſſed in the ſame way, three-fourths of an inch below below the firft; this being fecured alfo, the artery was divided between the two ligatures, and the needle being thus paffed through the cellular membrane, the thread could not flip from the artery, to which that membrane firmly confined it. The wound was united as far as it was poffible by the firft intention; the thread feparated upon the fifth day, and in feven weeks the wound was healed, and the motions of the limb reftored*.

But although this plan, as to the event, anfwered my expectations, yet a different mode of fecuring the ligature, fuggefted to me by my young friend, Mr. H. Cline, (fon to the furgeon) ftruck me fo forcibly for its fimplicity and fecurity, that I felt immediately difpofed to adopt it.

Before, however, it was ufed upon the living, I refolved to afcertain its effects upon the dead fubject; and finding that when the ligature was applied, I could not by throwing water into an artery with all my force difengage the thread from its fituation, I felt perfectly fatisfied of the fecurity which it afforded, and adopted the plan in the following cafe.

### Case III.

Henry Figg, aged 29 years, a farmer's fervant, of fo healthy a conftitution as not to recollect his having been confined by any ficknefs; in Auguft laft, whilft at work, felt a fevere pain at the back part of the right knee, which prevented his purfuing his labour, but ceafed when he became warm in bed; from that time to the month of December, he had frequent attacks of a fimilar kind; and he then firft obferved a fmall fwelling in his ham, in which he could not difcover any pulfatory motion; as it increafed it became pulfatory, and the pain and inconvenience which he fuffered, induced Mr. Martin, of Ryegate, (whofe patient he was) to fend him to me. On Wednefday, April 28, he was admitted into Guy's Hofpital, and, on the enfuing Monday, I performed the operation for Aneurifm in the following manner. An incifion being made on the middle of the inner part of the thigh, and the femoral artery expofed, the artery was feparated from the vein and nerve, and all the furrounding parts, to the extent of an inch, and an eyed probe, armed with double ligature, having a curved needle at each end, was conveyed under the artery, and the probe cut away. The ligature neareft the groin was firft tyed; the other was feparated an inch from the firft, and tyed alfo; then the

 needles

* Some curious and interefting circumftances took place during this lad's recovery; but as they do not relate to the immediate object before me, I have intentionally, in this account, avoided relating them.

needles were paſſed through the coats of the artery, cloſe to each ligature, and between them; the thread they carried was tyed into the knot of the ligature which had been already ſecured around the veſſel; and thus a barrier was formed in the artery beyond which the ligature could not paſs.

The wound was united by the firſt intention, except where the ligature projected; one of the threads ſeparated on the 14th, the other on the 15th day; and upon the 30th of May, the man walked acroſs one of the wards of the Hoſpital. On the 14th of June the wound was healed, and he ceaſed to ſuffer any inconvenience from the operation.

The annexed plate will explain the nature of this operation. One part of it ſhews the probe, armed with the ligature and needles; the other, the artery tyed at two points; the needle is ſeen paſſed through the artery, and the ligature which it carries is to be tyed to the thread hanging from the knot upon the veſſel.

Two queſtions will naturally ſuggeſt themſelves to thoſe who reflect upon this ſubject. Firſt, it may be aſked, If a ligature is liable to be thrown from the femoral artery, in the operation for Aneuriſm, why does not this accident happen in an amputation of the thigh, when the thread is applied only by means of a tenaculum? To this I would anſwer, that the artery in the two operations is under very different circumſtances. After amputation, the veſſel contracts to admit only ſo much blood as is neceſſary to nouriſh the part of the limb which remains; but when the operation for Aneuriſm has been performed, the arteries of the thigh muſt receive a ſufficient quantity of blood to reſtore circulation to all the parts below. Secondly, as this operation has been ſeveral times performed without the ligature having ſeparated, what was the reaſon of the accident in the caſe which I have deſcribed? The cauſe, I believe to have been this: that this patient, unlike the ſubjects of Aneuriſm in general, was of a ſtrong conſtitution, and did not appear to be reduced by previous indiſpoſition; in ſuch a perſon the impulſe of the blood at each pulſation of the artery, will be greater than the ligature can reſiſt, unleſs it is applied with a degree of force, which will endanger a laceration of the coats of the veſſel.

But, as the plan which was purſued in Caſe the third, removes, under the greateſt force of arterial action, the poſſibility of danger, and can in no inſtance be productive of the ſmalleſt riſk, it appears to me to deſerve to be generally adopted.

## Figures and Tables

**Figure f1:**